# Virtual carers for the elderly: A case study review of ethical responsibilities

**DOI:** 10.1177/2055207616681173

**Published:** 2016-11-28

**Authors:** Tom A Garner, Wendy A Powell, Valerie Carr

**Affiliations:** 1 School of Creative Technologies, University of Portsmouth, UK; 2 WeAreSnook, Glasgow, UK

**Keywords:** Machine ethics, digital avatar, virtual carer, elderly care, automated systems

## Abstract

Intelligent digital healthcare systems are becoming an increasingly considered approach to facilitating continued support of our ageing population. Within the remit of such digital systems, ‘Virtual Carer’ is one of the more consistent terms that refers to an artificial system capable of providing various assistive living and communicative functionalities, embodied within a graphical avatar displayed on a screen. As part of the *RITA* (Responsive Interactive Advocate) project – a proof of concept for one such virtual carer system – a series of semi-structured discussions with various stakeholders was conducted. This paper presents the results of these discussions to highlight data security, replacement of human/physical care and always acting in the user’s best interest. These three ethical concerns and designer responsibilities are identified as highly relevant to both individuals and groups that may, in the future, utilise a system like RITA either as a care receiver or provider. This paper also presents some initial, theoretical safeguard processes relevant to these key concerns.

## Introduction

Gains in life expectancy have caused the older population to increase significantly in numerous regions across the globe.^1^ The key question raised is how we can provide quality care to more people with increasingly stretched resources. In response to this, emerging technological developments in robotics and artificial intelligence systems with the intention to provide companionship and support to older and more vulnerable people are becoming a reality.^2^ Such technology has the capacity to improve the lives of older people by facilitating greater independence and creating more opportunities for social interaction.^3^ Implementation of this form of technology is, however, not without potential risk. In fundamental terms we are discussing the integration of autonomous systems into the homes of vulnerable people; systems that have the capacity to control most aspects of the home environment and potentially alter the experience of life for the user. Consequently, it is essential that this form of autonomous system accommodates a comprehensive ethics framework to protect its users.

**Figure 1.**
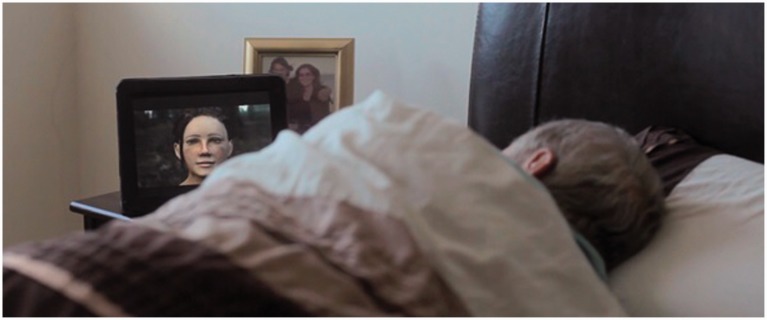
Screenshot from RITA concept video (http://rita.me.uk/demo/).

**Figure 2.**
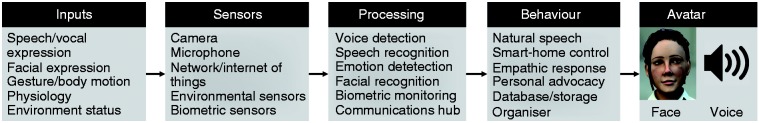
Basic flow graph outlining RITA’s structure.

The Responsive Interactive Advocate^4^ (RITA, see Figure 1 below) project developed a proof-of-concept system built around three primary components: (1) a high-resolution 3D human-like avatar that could support real-time conversation; (2) a data repository for storage and organisation of various forms of information pertaining to the user; and (3) an emotion detection and classification framework to enable the avatar to respond to affective input from the user. Figure 2 outlines the basic elements of the RITA system, in which various environmental inputs are detected by way of several sensor types; the information then processed to drive naturalistic behaviors of a digital avatar front-end. Implemented within the home environment, RITA would have the potential to influence user decisions with regards to various sensitive components of their lives, from healthcare to personal relationships. Ethical guidance in associated areas (primarily robotics) already exists but not yet within the specific context of non-physical, digital avatar-based systems. Consequently, this paper examines the concepts, debates and assertions surrounding contemporary ethical issues, to produce an original set of ethical considerations directly relevant to the RITA project and similar virtual carer endeavours.

Virtual Carers (VC) can be classified as ‘Ambient Assisted Living’ (AAL) technology; electronic and digital systems that are developed to provide a healthcare and wellbeing functions within the home environment. AAL systems often run on PCs and mobile/tablet platforms, and interact with everyday electric and electronic home appliances.^5^ Several AAL functions dictate a relatively significant level of system control over the user and their environment; these include: administering preventative treatment (ensuring medicines are taken, providing dietary advice, managing exercise programmes, etc.), providing assistance in the event of a health/wellbeing problem and tracking the user’s location within their home.^6^

Utilising a graphical avatar to represent the central interface of an AAL system is more commonplace as part of a further category of VC, the ‘virtual assistive companion’. Based upon embodied conversational agent technology,^7^ virtual companions typically consist of a human-emulating graphical avatar that utilises biometrics and/or environmental sensing technology to collect data that is then processed by way of emotion recognition architecture. This processed input, in conjunction with speech detection and vocal synthesis systems, supports natural dialogue, social interaction and, ultimately, an enduring and beneficial relationship with the user.^8^ Potential benefits of note include prevention of social isolation and motivational support in everyday activities.^9^

cA literature review addressing the ethical implications of integrating a VC such as RITA into people’s homes faces an obstacle in that very little research has been done that directly addresses this form of technology being employed for this particular purpose. As a result, parallels are drawn between much of the following literature and the VC context. The intention of this work is to compare the literature with the information obtained from a recently conducted series of structured ‘user consultations’ to reveal what key stakeholders feel are the most pressing ethical concerns and, where possible, present some potential approaches to managing these issues.

## Living with artificial systems

Much of the research concerning ethical issues within an assistive living context focuses upon robots rather than non-physical, digital avatars. However, many of the issues raised within such literature are nevertheless relevant to avatar-based systems because, although their visage is virtual rather than physical, both the nature of the system–user relationships and the underlying artificial intelligence processes remain comparable. First officially recognised in 2004,^10^ the term ‘roboethics’ has referred to guidelines for developing robots so that they may effectively cohabit with people.^11^ This cohabitation related to what Weng^12^ (p. 1919) describes as ‘the robot sociability problem’ that arises when social robots with autonomous functionalities interact with humans. Specifically, the capacity of the robots to elicit changes in the lives of the people they interact with is the primary source of concern. Scheutz^13^ asserts that the majority of ethical issues concerning robotics and autonomous artificial systems are yet to be conclusively resolved, implying that, as a culture, we are currently at a stage for discussion and debate, not yet prepared to ratify conclusive decisions. Scheutz elucidates the complexities of automated behaviour by way of moral dilemmas in which robot assistants will likely be faced with situations in which ‘no matter what they do, they are likely to cause humans pain and suffering’^13^ (p. 20). Here the intelligent system is not simply tasked with taking a course of action that avoids a negative outcome, but rather must assign some sort of value to each alternative outcome to determine the ‘optimal’ outcome. There is no perfect right or wrong. Whilst a VC system may not possess the physical presence of a robotic equivalent, its typical function of integration with home appliances and technologies gives VCs direct control over the user’s environment. Furthermore, as a socially communicative machine, a VC such as RITA is explicitly designed to impact upon the user’s affective state, and is likely to be presented with complex scenarios in which optimal behaviour is extremely difficult to determine, thereby risking the VC unintentionally causing emotional distress.

A recent European study exploring ethical issues relating to assistive living robots noted that the key concerns expressed by potential users were risks to dignity and the user’s autonomy, treating the user in a condescending manner and restricting the user’s executive control over both daily and long-term decisions.^14^ The issue of dignity is also addressed by Sharkey,^15^ who suggests that a ‘capability approach’ is required to support the wellbeing of the user. With regards to this approach, Sharkey outlines a range of functions for maintaining dignity, including: provision of physical security, supporting a wide range of experiences and expressions, supporting recreation, and encouraging freedom of sensory experience, imagination and thought. These items are what Nussbaum^16^ perceives to be essential requirements for individuals to possess a personal sense of dignity, and Sharkey asserts that any intelligent assistive system should aim to support these capabilities and address any characteristics of the system that may mitigate them. A VC such as RITA would, with appropriate integration, certainly have the capacity to support the above requirements for dignity. However, a problem arises when we consider that these requirements have the potential to be contradictory in practice (particularly with vulnerable users), with the VC being required to determine a trade-off between physical security and wider experience.

Coeckelbergh^17^ warns that a gap between expectation and reality in terms of what the user believes the system can do and its actual capabilities is a potential cause for concern. Sandoval and colleagues^18^ emphasise this concern, positing that people’s expectations are noticeably affected by fictionalised representations of robots in popular cinema, leading to a perception/reality mismatch. Many films appeal to people precisely because they offer an opportunity to explore these philosophical concerns.^19^ Infantilisation is a further concern raised in publications addressing ethical concerns for VC systems^3,14^ in which presenting older users with doll/toy-like artefacts, irrespective of their therapeutic application, is described as potentially dispiriting and encouraging of regressive behaviour by way of associating being elderly with being a child.^20^ In their review of ‘everyday’ ethics in care for older people, van der Dam and colleagues^21^ posit that communication with family, friends and colleagues is an essential freedom and that an automated, socially proficient machine may risk isolating individuals from such relationships by providing an immediate and constant alternative. Sharkey and Sharkey^3^ present eight primary potential ethical risks of integrating robots into the homes of older people: (1) reduced person-to-person contact; (2) increased feelings of objectification; (3) feelings of losing control; (4) loss of privacy; (5) limits to personal liberty; (6) deception; (7) infantilisation; (8) dangers associated with giving an individual the power to control robots. Many of these concerns relate strongly to Nussbaum’s^16^ requirements for dignity, but also extend to considering potential risk to individuals outside of the user, such as family, friends and healthcare workers.

## Data security concerns

The acquisition, processing and storage of information, much of it personal and sensitive, are central functions of a VC such as RITA. This positions data protection and security as significant factors affecting the wellbeing of the user. Wilkowska and Zeifle^22^ identify security and privacy as two primary discrete issues relevant to data protection within a digital health context, and regard an appropriate response to these issues as crucial to attaining high user acceptance rates when attempting to integrate digital healthcare technology into people’s homes. Whilst the concerns end users have regarding security and privacy may be instinctive, they are not without precedent, as the frequency of breaches to information security has remained high over the past 30 years.^23^ Concerns that personal information may be utilised by government or private organisations for monitoring or tracking purposes are prevalent.^24^ Providing information security is, however, a complex task, often requiring compromise between protection, flexibility and the often prohibitive costs.^25^

Abdulhamid and colleagues^26^ discuss cybercriminals’ intent on acquiring sensitive personal information, stating that they have been known to utilise increasingly sophisticated approaches that include hacking existing profiles or creating facsimiles of a user’s real friends, and concealing malware within messages or websites that have the potential to automatically adjust security settings, disable encryption protocols and expose personal data. This highlights the importance of user awareness and responsibility; specifically, to have an awareness of cybercriminal tactics and to engage with custom security settings for all aspects of the profile. Of particular note is their assertion that information that informs others of your location may be particularly compromising to security (e.g. increasing the risk of being burglarised if people are aware when you are not at home).

Wireless sensor networks have gained in popularity amongst both developers and users.^27^ Such systems can transmit data efficiently between the user, medical professionals and third parties with significant mobility, and are also capable of exchanging data between both local and remote locations. However, Al Ameen and colleagues^24^ state that wireless networks generate their own unique set of security vulnerabilities that include the potential for denial of service attacks (DoS: commonly referring to the act of overloading a system to disable its functionality), data modification (interception and alteration of data before sending the now false information to the originally intended recipient), eavesdropping (collecting data for malicious purposes such as identity theft), impersonation (generating a false user identification to abuse the service) and tracking the user’s location. Much like wireless networking, cloud computing (data is spread across multiple servers that are typically geographically distant from one another) provides improvements in reliability and efficiency but presents a set of new systems and interfaces potentially vulnerable to malicious attacks.^28^

Across the many social networking sites currently in operation, the quantity of personal information that is commonly requested (though is not always mandatory) as part of registration is substantial. Profile information typically includes photographs of the user, employment information, gender, age, marital status, hobbies/interests, location, education history, religious beliefs and nationality.^26^ Additional safety problems arise with regards to the complex and dynamic social norms experienced within online social communication. Binder and colleagues^29^ assert that, unless properly educated, users are at risk of disrespecting or offending (or being offended by) individuals or groups, potentially leading to social exclusion and possibly isolation.

With regards to social networking (as a relatively comparable system to the underlying data functions of RITA), Leitch and Warren^30^ provide a concise account of continuing data protection/security concerns: (1) the inclusion of third party applications limits the security of social media sites and, for users with such ‘add-ons’, not all exchanged information is guaranteed to be encrypted and therefore is susceptible to interception and data theft; (2) third party applications may be fraudulent and built expressly to access private information; and (3) the potential for users themselves to unintentionally alter their security settings and release private information. Leitch and Warren also identify security dangers with regards to harassment and stalking, defamation and disparagement (spreading false/misleading information, slander, ‘trolling’, etc.) and also vulnerabilities to malware attacks. Faisal and colleagues^31^ reinforce the notion of user responsibility with regards to privacy, as does Hoffmann,^32^ stating that ‘users of Facebook who have their privacy set to a custom setting are less likely to receive an attack on their profile’.

## Human emulation: Issues with giving virtual carers a human face

**Figure 3.**
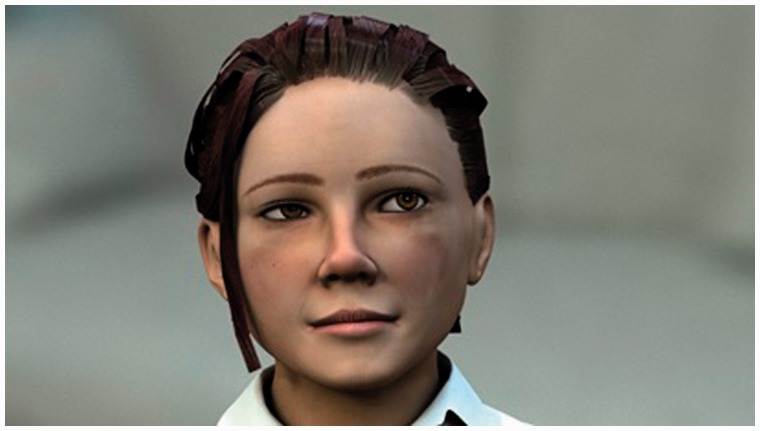
Fully rendered RITA avatar.

The RITA avatar utilises advanced modelling, motion-capture-based animation and high-resolution texturing to produce a highly detailed representation of a human (see Figure 3). Within a healthcare and wellbeing context there is very little conclusive evidence to support or refute the efficacy of human-emulating avatars over animal of other non-human characters. However, research has asserted that more realistic human designs are more likely to evoke a sense of competence and trustworthiness, an assertion that can be evidenced by noting that the majority of healthcare-associated avatar systems utilise a human avatar.^33^ However, with regards to ethical concerns, Sharkey and Sharkey^34^ note that being presented as ‘human-like’ by way of their appearance and actions in the interest of nurturing a relationship is itself a deception.

For a robot or virtual agent to infer humanity is essentially a lie, and Sparrow^35^ argues strongly that such deception is unethical, specifically because the proposed benefit of a relationship is dependent upon the user willingly engaging with an illusion. Sharkey and Sharkey^34^ acknowledge the presence of deception, but question assertions that such an act is unethical or damaging. Their primary argument is that processes of anthropomorphism and acting upon deception are commonplace outside the context of robotics. This argument is supported by Zizeck,^36^ who suggests that people are able to reconcile reality and illusion in a form of duality, simultaneously knowing that an item is inanimate whilst interacting with it as though it were living. This process is established in childhood studies^20^ (p. 283) but arguably also extends to adults when considering phenomena such as suspension of disbelief.^37^ In terms of the relationship between user and virtual carer, there is concern that the user is being required to invest emotionally in the system as if it were human when it is unlikely the system would be capable of reciprocating to such an investment in the same way a human could.^38^

Sharkey and Sharkey^34^ acknowledge several potential concerns associated with anthropomorphism of artificial intelligence systems. Users may feel a sense of duty or responsibility towards the system, prompting them to put its (perceived) welfare ahead of their own. Users could potentially be made to feel a further sense of infantilisation, particularly if the system (or something comparable) has visible function for children. An artificial companion with a human visage could present an enhanced problem regarding the social isolation concern (briefly outlined in previous section), simulating social communication so well as to limit user motivation for pursuing actual human social contact. It may also discourage others from interacting socially with the user, under the presumption they are superfluous now that the user has ‘their machine’. The potential for social risk in the design of a human-like digital avatar is a genuine concern, whilst the alternative approach of an animal or machine-like appearance carries issues with infantilisation (and is still at risk of deceiving the user and increasing social isolation as they instinctively anthropomorphise the character), and is possibly even more problematic. Attempts to construct a hi-fidelity, accurate digital representation of a human brings forth an additional ethical concern that relates to the ‘Uncanny Valley’ principle. First described by Mori,^39^ the uncanny valley asserts that, as artificial constructs reach ever-greater sophistication in their visual design, there is a significant drop in humans’ positive emotional response to them. Approximations of the human visage have the potential to evoke anxiety and discomfort. Avatars that find themselves positioned within this valley are also expected to inherently evoke distrust, as their design represents something that is received as both alive and non-living, an experience that can be distressing for many. Contemporary literature largely acknowledges this effect event even in modern, hi-fidelity avatars.^40^ An evaluative experiment by Tsiourti and colleagues^8^ revealed that older adults were generally accepting and responsive towards humanoid avatars as the front of a virtual companion system. They did, however, show preference towards more abstract, cartoonish designs as opposed to more realistic human representations.

Uncanniness does not, however, present a ubiquitous problem for virtual character appearance, and a recent study concerning video game non-player characters asserts that whilst uncanniness may still be a barrier to acceptance of the character, it does not limit the players engagement with the virtual world as a whole.^41^ When looking specifically at avatars that function within a role that is conditional upon trust, such as a carer, realistic human appearance presents some benefits. In a study by Riedl and colleagues,^42^ perception of a face as human significantly increased participants’ ability to predict trustworthiness. When comparing a range of ‘cognitive agents’ (automated systems that exhibited varying degrees of ‘humanness’, and included an actual human on the scale), de Visser and colleagues found that increased human quality of the agents also increased both trust predictability and appropriate compliance from the participants.^43^ With a focus upon older people, Chattaraman and colleagues^44^ posit that human-like virtual agents can increase participant trust and alleviate anxiety in an online retail context. Because a VC system such as RITA incorporates various functions that are dependent upon user trust (such as managing their home security, personal finances and medication schedule), this raises significant questions with regards to the graphical design of a VC, and whether the specific context of its use negates some of the above concerns that largely relate to recreational and therapeutic applications.

## Distrust of contemporary technology

Conditions that manifest as anxieties with regards to new technologies are relatively commonplace, to the extent that the phenomenon warrants its very own term: ‘technophobia’.^45^ It is easy to presume that the relative ubiquity of modern technology is indicative of a reduction in technophobia, but recent research has actually revealed the opposite.^43,44^ Furthermore, technophobia is not limited to vulnerable demographics, and wider population is also susceptible to its effects.^46,47^ However, research has indicated that autonomous robotic agents may not be a source of anxiety or distrust provided that the system design enables the user to clearly observe and understand the complete functionalities of the robot (see transparency, discussed later in this paper).^48^ The level of technophobia is likely to depend on both prior experience^49^ and the degree to which the system matches expectation and users’ understanding of current technology.^45^ Whilst direct user experience testing with VC systems such as the RITA prototype would undoubtedly provide more conclusive evidence in this matter, at this preliminary stage it seems appropriate that the conceptual design of such systems should incorporate procedures that elucidate the functionalities and processes of the entire system to ensure complete transparency of functions and processes.

As mentioned briefly in the previous section of this paper, trust is a central issue with regards to supporting the core functionality of a VC system. The user is required to have trust in the VC to the extent that they are comfortable with acting upon its direction. The ethical difficulty raised here concerns whether automated intelligent systems can be given the decision-making power that trust of the user affords them whilst simultaneously guaranteeing user safety. Law and colleagues^50^ discuss some of the difficulties in ensuring that the behaviour of an autonomous system is reliable when it is likely that at least a proportion of its input is unreliable, suggesting redundancy measures need to be in place to support the system in identifying such input and switching to an alternative information source. The question here is where the line should be drawn between autonomous, evolving functionality and structured response behaviours; the former risks unreliable and potentially unsafe VC actions whilst the latter severely limits the functionality of the system.

## Summary of literature findings and user consultation results

The following table brings together the above information on functionality and ethical concerns, summarising potential risks to user physical and psychological wellbeing that might arise in the implementation of a VC system in a real-world environment. Here the intention is to focus upon providing a comprehensive review of issues that pertain to VCs such as RITA. With regards to the ‘associated function’ column, RITA functionality is codified, the key for which is at the foot of Table 1.

**Table 1.** Relating primary functionalities of RITA to ethical concerns and potential solution.

Issue & associated function(s)	Specific concerns	Potential approach/solution
*Accessibility* **4, 5, 10**	Novel solution may be unintuitive/Users may be used to different systems / Large volumes of complex data and unclear charts may limit understanding / Wide range of functions and options may increase operational complexity / Novel interface difficult to operate	Utilise communication system (RITA can explain her own operation) / Communication and emotion detection could enable RITA to infer problems and engage responsive tutorials / interface design based on established systems / novel features tagged by system so RITA is ‘aware’ increased support may be needed in these areas
*Affordability* **1, 5, 7, 12**	Biometrics hardware not cost effective / High fidelity, real-time animations may require expensive hardware to run / Large databases may require expensive storage space	Exploit multi-use hardware to limit need for additional biometrics (e.g. camera for face and expression detection; microphone for voice detection/control)
*Autonomy* / *Personal liberty* **2, 3, 8–11**	User feels that they are losing responsibility for monitoring their own health and wellbeing themselves / User may not wish for their data to be uploaded without consent or may change their mind about what can / cannot be stored / Activity recommendations may reduce user’s sense of ownership over their own life / Users feel patronised by constant reminders and health advice	User-autonomy threshold system (Figure 3) / All automated processes can be identified and cancelled upon request / regular automation review (transparency failsafe) / machine learning for user preferences
*Deception* **1, 3, 6, 7, 13**	Human-like roles and appearance leading to suspicion that RITA is pretending to be human / Automated processes that are not fully disclosed and understood by the user may create suspicions of deception / Collecting any personal data on the user without their understanding and informed consent may create similar issues	Integrated reminders where RITA states she is a machine / transparency failsafe / option for non-human avatar appearance
*Attachment* / *Duty to the system* **1, 3, 6, 7, 13**	RITA as friend / advocate may lead to attachment / Withdrawal effects if RITA is taken away / Encouraging the user to behave in ways they feel are beneficial to RITA at a detriment to themselves	Variable contact-use time limits between RITA and user/regular appraisal of user independence / increase of user independence a core function
*Human carer impact* **3, 6, 8–13**	RITA functions may limit perceived value of human services / Users may wish to replace human carers with artificial system / Job market impact	RITA function responsive to human carer role / integration and support rather than replacement
*Fear / distrust of the system* **1–13**	Human-like appearance evoking uncanniness and discomfort / Innovative functions appear too futuristic for the user (too removed from their experience of the everyday) / Health and emotion-related functions too vital and personal – inherently instilling distrust	Option for non-human avatar appearance / interface design based on established systems / transparency failsafe
*Infantilisation* **1, 3, 4, 6, 11**	RITA seen as a toy / User could feel talked-down to / effect of reduced autonomy	Avoid ‘gimmicky’ functionality and design choices / RITA presented as a tool / increase of user independence a core function
*Intrusiveness* **2, 8-13**	Invasive biometrics could be disruptive and potentially upsetting / Consistent reminders could become an irritant Coaching and management advice and control could be disruptive and irritating / Recommendations and unrequested advice could become intrusive / constant presence of RITA limits option for solitude	Use integrated (contactless) camera and microphone where possible / machine learning (preferences) to limit unwanted advice / emotion recognition to infer user preferences non-intrusively / Variable contact-use time limits between RITA and user / ‘OFF’ switch
*Power* **11**	User may abuse the advocacy of RITA, using the system to inappropriately interact with third parties (unfairly monopolising their time and resources)	Problem-reporting facility (third parties may raise issues with RITA service support team) / emotion-detection to facilitate automated RITA interventions
*Privacy* **1, 2, 5, 13**	Third party sharing / Emotional and health status monitoring too invasive / Automated collection storing data the user does not want recording / data after death	Highly customisable privacy and monitoring settings / regular review / emotion-detection to infer preferences
*Data security* **2, 5, 8**	All personal data accessible from single point / wireless interception / cloud vulnerabilities / virus & malware risks / accidental security mistakes made by user	Biometric identification (facial/voice / etc.) / strong encryption / supporting secure use central part of system functionality
*Reduced human contact* **1, 3, 4, 6, 13**	Distraction from physical or community-based interaction / User overly reliant on conversation with RITA, discouraging them from pursuing personal relationships	Increase of user independence a core function / person-to-person contact actively encouraged by RITA
*Safety* **3, 7, 8, 9, 10**	No human failsafe should an accident occur / data malfunctions potentially harmful / Emotion interpretation mistakes could cause emotional distress	‘OFF’ switch and user-override facility / physiology and environment monitoring for intelligent threat-detection / option to feed monitoring to human (carer)
*Third party communication* **1, 3, 6, 10, 11**	RITA may accidentally damage relationships with friends and family whilst acting as an advocate	Regular review / emotion-detection to infer preferences
**RITA Functionality key: [1]** Appearance (human-like character) **[2]** Automated data collection **[3]** Carer role **[4]** Communication device **[5]** Database / storage **[6]** Emotional / wellbeing support **[7]** Emotion detection / interpretation **[8]** Environment / home monitoring **[9]** Exercise coach **[10]** organisational support **[11]** personal advocacy **[12]** physiology monitoring / biofeedback **[13]** real-time conversation (voice detection / speech synthesis)		

Conducted by the design agency *WeAreSnook* (http://wearesnook.com/), a ‘user consultation’ programme of semi-structured group discussion sessions collected qualitative information from 13 individuals comprising a reference group (all respondents aged between 54 and 81), eight voluntary groups (totalling 85 individual participants), six private care providers (133 respondents) and five statutory service providers (seven respondents). This broad range enabled pooling from a broad range of opinions and also indicated significance in the more consistent findings. Across the different groups questioning was largely uniform, with the only variation within the user reference group, who were asked to identify themselves as having either a lay or professional interest in RITA and also to state their profession if applicable. The core areas where input was requested were: (1) Comments on general concept, development of the visual design/avatar, the system’s support functionality and the scripts that would form part of RITA’s speech; (2) What is exciting about the concept; (3) Identify ideal functions; (4) Note any concerns and reservations. For the purposes of this paper it is the final question that we are focussing on here.

**Figure 4.**
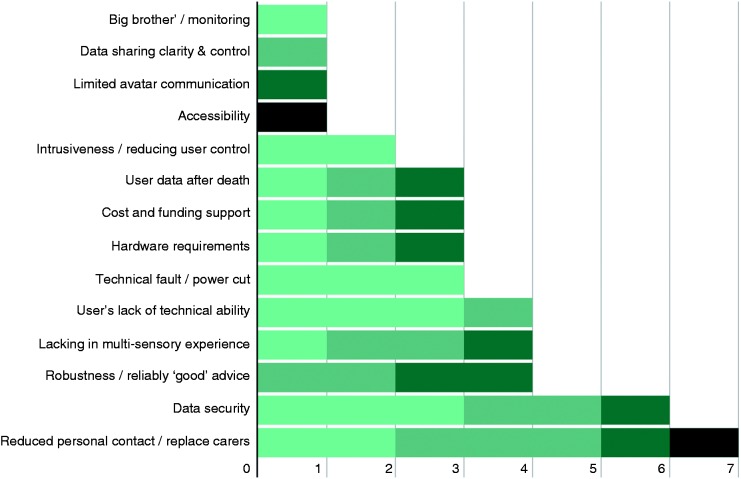
Results of discussions across all groups.

The chart above (Figure 4) presents an overview of the feedback we received from the various groups. As is highlighted in the above, concerns regarding data security, user safety and reduced personal contact rated as consistently high priorities. Expanding upon these, respondents were concerned that having a single point of access for a comprehensive set of (likely sensitive) data was a significant risk. This worry was not limited to system robustness and implementation of strong security, but also included user action concerns, citing hypothetical scenarios in which users accidentally released their own data through either difficulty using the system or through deception at the hands of cybercriminals. Another related concern was that it was unclear how user data would be managed following that individual’s death. User safety encapsulated issues of RITA’s decision making, specifically questions regarding how an automated system intervening and making decisions that directly impact upon the user can be designed to ensure that RITA always acts in the best interest of the user. Reduced personal contact largely related to three common issues: that automated systems such as RITA could replace human carers; that a digital service is inherently sterile and lacks the power to provide multi-sensory experience (RITA cannot actually give you a hug); and that the service could become ‘too successful’ and discourage users from seeking human contact. As development on RITA progresses, these results help focus attention on the ethical concerns that matter most to the stakeholders that will actually be interacting with such a system. This is not to say that the other concerns presented with the literature are irrelevant, but rather that data security, user control/safety and reduced human contact require transparent solutions to encourage stakeholders to embrace the overall concept.

## Managing data security

With both the literature and the results of our user consultation positioning data security as a top-tier concern, the following section presents some initial approach ideas and recommendations relevant to this issue. Establishing access to private information by way of identity verification reveals a range of potential options. From the (now seemingly archaic) password to combined biometrics, the number of options for identification is increasing. Research has posited automated facial structure examination combined with voice detection,^51^ iris analysis^52^ or the use of an electrocardiogram to measure heart activity.^53^ Umphress and Williams^54^ have suggested that the mean keystroke latency (average time latency between strokes on a keyboard) could potentially discriminate between people and be utilised for identification purposes, a notion that Cho and colleagues^55^ later revived, recommending that keystroke dynamics in conjunction with traditional passwords could significantly increase security dramatically. For the purposes of RITA, any forms of biometric identification that require direct contact with the user are less than ideal because they are likely to be perceived as invasive. Conversely the possibility of employing non-contact biometrics for automated user identification, reducing the need for users to generate and remember complex high-security passwords, is certainly an attractive one.

Passwords have enjoyed an extended tenure as principal method for user identification. However, the developing sophistication of hacking techniques and related technologies have required the humble password to develop somewhat in recent years.^56^ Nelson and Simek^57^ assert that most passwords consist of around eight characters and that contemporary hacking techniques are capable of cracking passwords of that length within 2 hours. In comparison, a 12-digit password would (with present technology) take more than 17,134 years to break. Nelson and Simek^57^ argue that the length of your password does not only determine how easily your data could be hacked, but also how likely you are to be attacked at all, as cybercriminals will characteristically target more vulnerable systems. Their paper also suggests a variety of password-related approaches to protecting personal data. Passphrases are recommended, specifically those that are personally significant and can be easily remembered but are also of significant character length to be secure. Combining upper and lower case plus integrating numeric values and symbols is also encouraged though, it is acknowledged that this can limit the ease with which the password can be recalled.

One further approach to identification for access to data comes from one existing approach software developers have employed as a means of protecting their intellectual property; namely software protection keys. Constructed much like a USB flash drive, protection keys are programmed with cryptographic protection (an inbuilt product code) that, when inserted into a computer, can activate software processes and decrypt information.^58^ Whilst Nelson and Simek^57^ acknowledge the high-security potential of this approach, they also question practicalities, specifically that the device can be troublesome to operate, easily lost, can be stolen and is difficult to replace.

Awareness and personal responsibility is often viewed to be the one of most effective approaches to ensuring security and privacy online. This assertion is supported when considering the research that has attempted to combat privacy and security issues through direct education of the user. Cetto and colleagues^59^ developed *Friend Inspector*, a serious game intended to increase awareness of privacy control options. This largely supports the assertion that directing attention towards the actual user, in terms of raising their awareness and motivating them to actively and continuously engage with security options, is one of the more effective means of protecting them and their digital information. A high level of user control is also raised as an important element. This suggests that RITA should provide users with a high degree of control over precisely (to the individual) who has access, what they have access to, and under what circumstances access is granted. Furthermore, RITA has a responsibility for maintaining awareness of, and engagement with, all aspects of the security features built into the system.

A fully customisable access system, such as those implemented within comparable data-holding systems (such as Mydex (personal data storage – https://mydex.org/), Facebook and Tyze (online care network tool – http://tyze.co.uk/)) reveals the following access determiners that could be implemented into a VC system: (1) *Who* – which individuals have access; (2) *What* – the precise pieces or groups of information that can be accessed; (3) *Where* – restrictive access with regards to location (e.g. the user may enable a third party to access certain files when they are local (in the presence of the user, via the user’s RITA device) but not remotely); (4) *When* – restrictive access with regards to time, enable third parties to only access certain information at designated times; (5) *How* – the user has control over third party authentication (e.g. requiring them to use multiple passwords, biometric identification, confirmation links within emails, set questions, etc.), enabling them to place multiple layers of security over more classified material; (6) *Why* – restricted data functionality (e.g. files (images/music/etc.) may only be available to use directly within the RITA system to some third parties whilst others may be allowed to download and take them away).

## Further steps

Existing research has already published guidance for the ethical evaluation of assistive technologies^60^ but there is currently very little directly addressing ethical concerns specifically in the context of virtual carer systems for older people. Sharkey and Sharkey^3^ note that the human rights laws within the UK focus primarily upon civil and political rights as opposed to economic, social and cultural rights. As a result, there is a significant lack of legislative protection for older people. They further argue that comprehensive legislation with regards to artificial systems could generate enforceable guidelines with regards to the design and implementation of such systems. Such guidelines include fixing the maximum duration for which the system and the user are allowed to interact between periods of human contact and integrating a permission failsafe in which the VC system must always gain consent before interaction. Sharkey and Sharkey^3^ also advocate the use of detailed consultations with elderly people, specifically the individual for which the system is being provided, to ensure that the system is comprehensively bespoke. Most encouraging (with reference to VC systems such as the RITA concept) is their assertion that many ethical issues may be successfully mitigated by way of a ‘value sensitive’ design approach – essentially a series of sub-process modules within the overall system that accommodate ethics as a preventative measure. This resonates greatly with the user consultation discussions that repeatedly voiced concerns over how RITA could be assured to always act in the best interest of the user.

In a recent paper by Ishak and Nathan-Roberts,^61^ transparency and feedback are presented as key solutions in terms of engendering trust in robot carers for older people. Transparency refers to systems that clearly display their functionalities within the architecture, enabling the user to easily understand the system, and is separated into four categories: *design transparency* (appearance must reflect function, must be communicable at all times), *reliability transparency* (must be able to evaluate own reliability and communicate this to the user) and *goals transparency* (must be able to demonstrate understanding of user’s goals and be clear regarding how its actions will help achieve those goals). *Feedback* refers to confirmation given by the system regarding its actions (task completion, success/fail, etc.).

**Figure 5.**
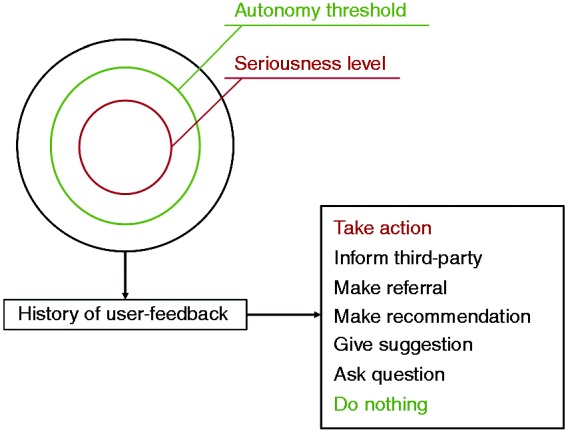
‘Autonomy threshold’ module design for the RITA virtual carer.

Research also suggests that VC systems should make deliberate effort to assess a user’s feelings of discomfort and concern to generate relevant moral questions that may not yet have been addressed.^21^ We can relate this to two pivotal issues: maintaining autonomy of the user (avoiding them feeling a lack of personal responsibility and control over their everyday lives) and intrusiveness. These are arguably inherent problems associated with artificial care because part of the system’s functionality is often to advise, encourage and potentially question the user, or even intervene in certain scenarios. Such an issue could potentially be managed by way of comprehensive user-control settings alongside sophisticated machine learning in which the system compiles and analyses user input to provide a more individualised response in future scenarios. For example, should the user feel that the support of the VC is becoming too aggressive and is intruding upon their personal liberty, the system (by way of biometric sensors and an affective intelligence framework) could detect this discomfort, engage the user to clarify their wishes and update the behaviour framework accordingly. Figure 5 presents an initial design outlining the structure for such a system module. Here the framework considers seriousness, autonomy and personal history to determine the particular form of action the VC will carry out. Forms of action have what we describe as an ‘assertiveness range’ from low/passive (e.g. do nothing) to high/assertive (e.g. take action), with various intermediate levels allowing for a more nuanced response system.

As can be exemplified in the RITA prototype design (Figure 5), a VC could consider the relative ‘seriousness’ of the issue (e.g. what piece of music will be played to wake the user in the morning = low seriousness; whether to embark on a course of experimental medications = high seriousness). If the serious rating exceeds the autonomy threshold, the VC would intervene in some way. By way of machine learning processes, the system’s interpretation of seriousness would be modulated by the user’s feedback history, enabling it to respond more appropriately to specific issues in a way more relevant to the individual user.

With regards to accessibility, it is clearly vital to consider the capabilities of the target demographic to comfortably operate and engage with the system. The development of a VC such as RITA should therefore strongly reflect popular and prevailing control mechanics and interface designs. Issues regarding economic impact (specifically the concern that VCs would replace human carers) should be managed on two fronts. Firstly in the development of the system itself, the core functionality and communication architecture of the RITA system should incorporate and facilitate direct interaction between the user and other humans. This could be realised in various ways, including: (1) the VC actively encouraging relationships with humans; (2) functionality to include helping the user select and acquire a new carer; (3) the system self-acknowledging its own limitations and advising on ways in which a human carer could provide additional benefit.

Regarding the third and final key issue, you could be forgiven (particularly if reading recent headlines) for feeling that technology has something of a hostile takeover agenda, carrying out duties that make certain human roles superfluous. This has been an ongoing fear for decades and has been argued against for an equally long time, the primary counter-position being that technology ‘is a human tool, not a replacement’.^62^ Supporting and explicating this argument is central to addressing this concern, and VC development should be no exception. It is therefore vital that VCs are envisaged as integrated tools for connecting users with healthcare professionals (and the wider community) and that this forms a foundation of its design, with VC intervention only provided as a means of enhancing human care or for times when such care is simply not immediately available. A system like RITA should endeavour to make healthcare workers’ practice more efficient by managing data and administrative duties to free up more time for professionals to deliver the aspects of care that are currently far beyond a machine’s capability. Furthermore, this concern highlights the importance of developing the VC as an active means of encouraging personal contact, with core functionalities (such as communication software, time-management/reminder support and web integration) that make social contact easier to arrange and keep the user updated with profile-matching social events and activities that may be of interest.

Overall it seems clear that the ethical landscape in which VCs sit is one of optimism, and that a considered and managed development strategy will facilitate a VC system that offers genuine lifestyle value without compromising the physical or psychological wellbeing of its users. Developing a complex system for use within an even more complex working environment certainly supports any assertion that a perfect system is an impossibility. Nevertheless, development strategies must strive towards the highest standards of safety and responsibility (particularly when we consider the general vulnerability of the target demographic). It could be argued that a VC system such as RITA should be custom-built to an individual user’s precise specification, though there are obvious financial implications for this approach and a user’s needs may evolve, making even a bespoke VC unresponsive to individual requirements following extended use. Consequentially a system that is adaptive and capable of evolving significant portions of its operating in line with user-demands is an ideal outcome. Such adaptive power would furthermore go a long way towards addressing the difficulty in reconciling some of the more contradictory issues (ensuring safety against promoting wider experience, providing emotional support against reducing motivation for human contact, etc.) by reacting to user input to prioritise a single preference within a conflict. Where an adaptive system would be limited, however, is when considering the initial ‘default’ settings (presuming the system was not built from scratch) and the extent to which the system can adapt to user input – relating to the complex question of whether an individual is, under all circumstances, the best authority regarding their personal decisions. Ultimately this is likely to mean that the VC system design, as it pertains to the above issues, is unlikely to be determined entirely at a consumer or company level, and must instead be informed by broader ethical frameworks and codes of practice.
